# Non-visible disability in the medical curriculum: what medicine overlooks, patients inherit

**DOI:** 10.3389/fmed.2026.1701393

**Published:** 2026-04-21

**Authors:** Waseem Jerjes, Azeem Majeed

**Affiliations:** Department of Primary Care and Public Health, Imperial College London, London, United Kingdom

**Keywords:** diagnostic overshadowing, diagnostic uncertainty, health inequalities, hidden curriculum, medical education, non-visible disability, patient safety, reasonable adjustments

## What we teach and what we miss

Medical education teaches more than biomedical knowledge: it also teaches what counts as credible evidence, which narratives are treated as diagnostically meaningful, and how uncertainty is handled in everyday practice (1). For clinicians in training, problems anchored to imaging or blood tests can feel safer to name and act on, while presentations with non-linear, fluctuating, or primarily subjective symptoms demand higher tolerance of uncertainty and more deliberate listening.

This article aims to make the hidden curriculum around non-visible disability explicit and, drawing on current literature and policy, to propose practical educational and system-level levers for clinicians, educators/curriculum leads, and system leaders—clinical reasoning habits, curriculum and assessment design, and reliable delivery of reasonable adjustments—to reduce diagnostic harm and inequities in access. It is intended for clinicians, educators and curriculum leads, and system leaders responsible for designing, recording, and delivering reasonable adjustments in routine care.

## Definitions and scope

In this article, non-visible disability refers to long-term neurodevelopmental, neurological, sensory, cognitive, pain, fatigue, or autonomic differences that shape daily functioning and access to care but are not reliably apparent in brief clinical encounters. Some are formally diagnosed; others remain under-recognized, late-diagnosed, or inconsistently recorded. We use the term hidden curriculum in its classic sense: the unspoken values, norms, and heuristics absorbed through training and workplace culture that shape what clinicians notice, legitimize, and act on beyond the formal syllabus (1). Diagnostic uncertainty is not the focus of the paper, but it is a key mechanism through which non-visible disability is missed, minimized, or mismanaged. This article does not present original curricular outcome data; rather, it offers an evidence-informed opinion that synthesizes current literature and policy to define an under-recognized educational problem and propose practical directions for curriculum and system improvement.

This argument also draws on social and social-relational models of disability: disability is produced not only by impairment or difference, but by the interaction between a person and environments, institutions, and norms that determine access, credibility, and support. From a social model perspective, non-visible disability is often made disabling by clinical pathways that prioritize visible, easily measurable cues; as a result, responsibility is placed on patients to disclose, justify, and repeatedly negotiate adjustments to access appropriate care.

Autism, dysautonomia, chronic pain, chronic fatigue, and sensory processing differences are common examples of non-visible disability that can be missed, particularly when symptoms fluctuate or do not map neatly onto a single test result. The familiar story is the late-presenting young adult who is exhausted and cognitively foggy, told repeatedly that “tests are normal,” and leaves without an explanation, a plan, or a clear reason to return. For many, this is not merely frustrating: it can be invalidating and destabilizing, laying the groundwork for distrust, avoidance of care, and, in some cases, experiences that patients describe as medically traumatic (1).

This pattern stems more from system design than individual intent. Needs that are obvious and externally legible (ramps, signage, step-free routes) are more likely to be anticipated and built into services. Needs that are less visible (quiet space, flexible waiting, paced communication, predictable sequencing, sensory adjustments) are often not considered at all unless a patient discloses them repeatedly and a clinician knows how to translate them into practical adjustments. What is seen is easier to standardize; what is variable is easier to miss (2, 3).

The consequences include delayed recognition, damaged trust, and avoidable use of healthcare through repeated re-attendance and low-value investigation. Autism is now diagnosed in approximately 1% of British adults, yet adult presentation can be subtle, masked, or misattributed, particularly when individuals have developed compensatory strategies over time (4). The difficulty of recognition is not confined to non-specialists: qualitative work has reported that autistic psychiatrists themselves may face barriers to recognizing autism in themselves, illustrating how easily autism can be missed without explicit training and a neurodiversity-informed lens (5, 6). In myalgic encephalomyelitis/chronic fatigue syndrome (ME/CFS), the UK National Institute for Health and Care Excellence (NICE) explicitly cautions against equating normal routine tests with absence of disease, underscoring the need for careful clinical reasoning when diagnostic certainty is limited (7). Similar dynamics apply to ADHD, dyspraxia, and sensory differences, where functional impact may be substantial even when traditional biomedical markers are absent.

Emerging educational evidence suggests that change is possible, although formal evaluation of curricular efficacy in relation to non-visible disability remains limited. Early empirical work indicates that teaching shaped with disabled people and led with learners can improve learner confidence and sensitivity in disability-relevant clinical skills (8). Extending this approach to non-visible disability requires making the hidden curriculum explicit: it shapes what clinicians learn to treat as “signal”, and what they learn—often unintentionally—to treat as “noise”.

## Non-visible disability in systems built for the visible

We often promote inclusion in principle, but in practice services prioritize adjustments that are easy to standardize, audit, and visibly demonstrate.

In the UK, the Accessible Information Standard (AIS) provides one structured example of how health systems can identify, record, flag, and meet communication needs in practice—for example by offering large-print or easy-read materials, providing an interpreter for British Sign Language (BSL), or using an agreed communication method. Although the AIS was developed primarily with sensory impairments in mind (including hearing and vision loss), its central aim—supporting effective communication and equitable access to services—also applies to people whose needs are less immediately visible, such as those requiring additional processing time, a quieter environment, or alternative modes of communication. In such cases, consistent documentation and proactive adjustments are essential to prevent avoidable barriers being recreated at each contact. However, implementation has been patchy, and reforms have been slow to translate into meaningful improvements for people with non-visible disabilities (9, 10). Although the specific policy tools vary internationally, comparable barriers arise in systems where documentation is fragmented, appointment structures are rigid, cultural norms make disclosure difficult, or reasonable adjustments depend on individual clinician discretion rather than routine pathway design.

The NHS also implemented the Reasonable Adjustment Flag (RAF). This is an electronic flag in a patient's record that documents agreed adjustments—such as provision of a quiet waiting area or flexible appointments—and is intended to communicate them across services. Theoretically, that means that patients don't have to explain their needs repeatedly at contact after contact. But without incentives, auditing, and defined clinical ownership, the system is inconsistently implemented. Visible adjustments are most likely implemented, but invisible ones become optional (11, 12).

Although this paper uses UK policy examples, the underlying problem is not country-specific. Across healthcare systems, cultures, and legal frameworks, non-visible disability is often inconsistently recognized, variably legitimized, and unevenly accommodated in routine care. While the route to exclusion differs between settings, barriers commonly arise when support depends on patient disclosure, clinician recognition, fragmented documentation, or local service discretion rather than being embedded into pathway design. Legal protections may establish duties to make reasonable adjustments, but translating those duties into sensory-friendly access, flexible waiting, pacing adjustments, and reliable continuity remains inconsistent (2, 11, 12).

Data systems widen the gap further. What is poorly coded is poorly counted—and what is not counted is rarely addressed. National health inequalities dashboards do not currently list hidden disability as a priority, so outcomes for these groups remain largely invisible to performance monitoring (13). Even in long-established priority areas, clinicians often report uncertainty about coding (for example in SNOMED) and about the accuracy of disease registers. Hidden conditions are even more likely to be under-identified (14).

Patient experience reveals the cost. Many autistic people report mainstream waiting rooms as intolerable and describe the importance of quiet space, predictable sequencing, or the option to wait elsewhere (15, 16). When such adjustments are unavailable, the result is not simply dissatisfaction but avoidance: appointments are deferred, canceled late, or never booked in the first place. The missed consultation is not coded, not audited, and not visible to dashboards—yet it may represent the highest-risk form of unmet need. This creates a self-reinforcing cycle: non-visible disability remains under-recorded, under-measured, and therefore under-resourced, while patients accumulate experiences of dismissal that further reduce willingness to return (1, 17, 18).

The disparity between the way hidden and visible disabilities are managed within everyday clinical practice is illustrated in Table 1.

**Table 1:** How visible and hidden disabilities are treated differently in clinical practice.

Aspect of care	Visible disabilities (mobility, sensory loss)	Non-visible disabilities (autism, chronic fatigue, dysautonomia, chronic pain)
First impressions	Needs legitimized immediately by physical cues	Needs can be missed when symptoms are fluctuating or not easily measurable; presentations may be misattributed under time pressure or uncertainty
Service adjustments	Physical access (ramps, signage, interpreters) increasingly routine	Adjustments such as quiet waiting rooms or pacing-aware consultations remain rare and *ad hoc*
Data visibility	Clearly coded, tracked in audits and inspections	Poorly coded, rarely measured; “what is not counted is not resourced”
Professional habits	Seen as straightforward to accommodate	Diagnostic overshadowing and premature closure more likely; stigmatizing shorthand can emerge (“non-compliant,” “difficult consultation”) unless explicitly addressed in training
Patient experience	Some frustration with patchy provision, but needs usually recognized	Frequent reports of disbelief, stigma, and avoidance of services; risks to safety and trust

Evidence sources: Concepts and examples in this table are informed by literature on the hidden curriculum (1), disability-related health inequalities (4, 18), diagnostic overshadowing (16, 17), autism and primary care experience (15), learning disability registers and coding gaps (14), and system-level adjustment mechanisms including the Accessible Information Standard and Reasonable Adjustment Flag (9–12).

## From hidden curriculum to diagnostic harm

The hidden curriculum influences outcomes as well as attitudes. Under time pressure, clinicians learn heuristics that reduce cognitive load but can distort care: “normal tests mean nothing is wrong,” “vague symptoms mean anxiety,” or “changing symptoms mean poor adherence”. These shortcuts are often learned as survival strategies in short consultations, but they can cause harm when the patient's main diagnostic signal is functional impact and narrative detail rather than a single objective marker (19). Compared with presentations anchored to a single objective abnormality, these encounters place greater weight on narrative, pacing, and context, so structural barriers and stereotype-driven assumptions have more opportunity to shape what is heard, coded, and acted on.

The risk is compounded by intersectional barriers: patients with non-visible disability who also face racism, poverty, language barriers, or gendered assumptions may be even more likely to be disbelieved or misdirected, widening inequities in diagnosis and access (20, 21). Evidence shows that implicit clinician attitudes intersecting disability and race affect beliefs about patients' behaviors and quality of life, and population data demonstrate that Black and Hispanic adults with disabilities experience greater disparities in access to care than those without disabilities, consistent with intersectional disadvantage frameworks in health equity (20–22). For example, autistic people report barriers to timely recognition and appropriate access, and patients with overlapping disability and mental health labels are at greater risk of diagnostic overshadowing in acute care (15–17).

Patients are often forced into narrow diagnostic boxes, given labels that reflect clinical uncertainty more than their true condition. In the short term, this leads to delays, missed diagnoses, fragmented care, and growing mistrust. Over time, the impact is felt at system level: repeated attendances for unresolved symptoms, cascades of low-value investigations, and polypharmacy that manages distress or impairment without resolving underlying drivers or functional needs. Evidence supports this system-level pattern: persistent medically unexplained symptoms are over-represented among high users of healthcare and are associated with higher healthcare costs, reflecting repeated investigation and re-attendance when diagnostic clarity and effective access adjustments are lacking (23, 24). This reinforces why curriculum attention to uncertainty tolerance, narrative signal, and adjustment literacy has implications not only for equity but also for system efficiency (23, 24).

One mechanism is diagnostic overshadowing: the misattribution of new or complex symptoms to a pre-existing label (or to “anxiety” or “functional” explanations) without sufficient reassessment, leading to compromised care (16, 17). In non-visible disability, this can operate in two directions: symptoms may be dismissed because they do not fit a visible biomedical pattern, or autism/ADHD traits may be missed because existing labels are treated as sufficient (6). Documentation can unintentionally harden uncertainty into identity: once “medically unexplained” enters the record without a parallel plan for review, it can shape subsequent decisions about referral thresholds, analgesia, and the seriousness with which symptoms are heard (16, 17).

These are not issues of etiquette; they are issues of safety. When patients feel dismissed or stigmatized, they often delay re-attendance until a crisis forces contact, by which time trust may be fragile and prior management plans may not have been feasible or appropriate. Patient experience is therefore not a “soft” outcome but a contributor to safety behaviors, continuity, and timely escalation (18). In non-visible disability, where functional history and narrative often carry much of the diagnostic signal, the quality of listening becomes a clinical skill with direct implications for diagnostic accuracy, shared planning, and ongoing engagement (5).

When training and systems privilege what is most measurable, clinicians can be unintentionally steered toward managing what can be counted rather than what most impairs function. The remedy is not more compassion alone but better clinical and educational design: explicit training in uncertainty tolerance, narrative skill, and the practical translation of need into documented adjustments.

## Unlearning the hidden curriculum: what clinicians can do now

If the hidden curriculum teaches us to prioritize what is visible and measurable, then unlearning it must be intentional, practiced, and supported by the system.

At the practical level of daily clinical work, Table 2 summarizes three action steps that can make non-visible need easier to recognize and act on, and shows how these can be operationalised so adjustments are reviewed and delivered consistently across contacts (11, 12).

**Table 2:** Practical action steps to recognize and deliver reasonable adjustments for non-visible disability in routine care.

Action step	Why it helps	Example wording	When/where in workflow	How to operationalise (so it happens reliably)
1. Brief diagnostic & management pause	Reduces premature closure when uncertainty is high (19)	“What might routine tests miss here, and what would change my plan today?”	Before finalizing assessment/plan	Embed in supervision prompts and consultation templates
2. One enabling question early	Surfaces sensory, pacing, and communication needs early, before time pressure escalates and shared understanding is lost	“What would make this appointment easier for you?”	Early in consultation (or pre-visit questions)	Add to triage/review scripts; normalize as a routine question
3. Record adjustments visibly and trigger delivery	Documentation supports continuity, but must be paired with prompts/enforcement to avoid reliance on individual memory (11, 12)	“We'll record your agreed adjustments so staff can support them each time.”	Booking/check-in/EHR; waiting area; consultation	Use the Reasonable Adjustment Flag and/or a visible alert field; staff training; a check-in checklist/prompt requiring staff to confirm recorded adjustments have been reviewed and implemented; routine monitoring (audit of adjustment review rates and patient feedback on whether needs were asked about and met) (11, 12)

## What education and assessment should change

At present, disability teaching in medical curricula is often uneven and may focus more on visible impairment, legal awareness, or general communication principles than on the practical recognition and management of non-visible disability in real clinical encounters. This gap spans undergraduate teaching, postgraduate assessment, and continuing professional development: learners may encounter disability as a professionalism topic without being trained to recognize non-visible disability as a diagnostic, access, and safety issue. Curricula can make the invisible visible by teaching non-visible disability as a core clinical and professional competency rather than an optional “inclusion” topic. OSCEs and workplace-based assessments should include scenarios where the key signal is functional history (post-exertional symptom flare, dysautonomia, sensory overload, communication fatigue), and credit should be awarded for pacing, clarification, shared planning, and safe follow-up rather than for premature certainty (8). What remains underdeveloped in many curricula is not only content but assessment emphasis. Learners may receive some teaching on disability, communication, or professionalism yet still complete training without being assessed on adjustment literacy, diagnostic overshadowing, sensory burden, pacing of consultations, or the safe management of uncertainty when symptoms are fluctuating and not easily measurable.

Training should also explicitly build uncertainty tolerance as a professionalism capability, not only by normalizing uncertainty but by teaching clinicians to continually broaden the differential diagnosis and to discuss uncertainty openly with patients. This can be supported through structured diagnostic and management pauses and brief equity reflections (“Who is being disbelieved, and why?”), helping to reduce avoidable reasoning error under time pressure (5, 19).

System levers can support this cultural shift and make it teachable in routine training. The Accessible Information Standard and the Reasonable Adjustment Flag should operate as live prompts at the point of care, with clear ownership, audit, and feedback loops when recorded adjustments are not delivered (9–12). Embedding these prompts and feedback mechanisms into staff induction, supervision, and workplace-based learning helps trainees and clinicians learn that identifying and delivering adjustments is a routine safety behavior, not an optional extra. Commissioning and provider contracts can also specify practical access expectations—quiet space where feasible, flexible waiting, predictable sequencing, and pacing-aware appointments—so that adjustments are normalized rather than treated as exceptional. This matters because access is not only a rights issue but a safety issue: when care becomes intolerable, patients disengage, and risk accumulates out of sight.

Further curriculum evaluation is therefore needed. Future work should examine whether teaching on non-visible disability changes not only learner confidence but also observable clinical behaviors, including whether clinicians ask about access needs, document reasonable adjustments, avoid premature diagnostic closure, and create safer follow-up plans under uncertainty. Evaluation should also consider patient-reported experience, whether documented adjustments are actually delivered in practice, and whether these changes improve access, trust, continuity, and use of care (8, 18).

Professional regulation is also an education lever, because it sets the competencies that curricula must teach and that clinicians must evidence and maintain over time. Regulators can support this by infusing latent disability expertise into graduate outcomes and revalidation, and by incorporating patient experience with greater weighting into quality dashboards (18). This matters because patient experience is not merely satisfaction—it is one marker of safety and outcome, particularly where access barriers lead to disengagement. In this framing, “unlearning” becomes a formal part of professional development: it starts once clinicians recognize what training and assessment systems have implicitly taught them to disregard.

## Professional identity: seeing what is not obvious

Recognizing the possibility of hidden disability is a matter of sound clinical reasoning, particularly when symptoms persist despite “normal” tests. However, responding appropriately—taking symptoms seriously, collaborating with patients to agree reasonable adjustments, and focusing on outcomes that are most meaningful to the person—is a core aspect of professionalism because it reflects respect, equity, and shared decision-making, and helps meet duties to make reasonable adjustments (2, 3, 18).

Professionalism in this domain is not a matter of abandoning evidence or clinical reasoning; it is how clinicians communicate and act when evidence is incomplete, the presentation is non-linear, and the patient's functional narrative is central. In such encounters, professional identity is expressed through epistemic humility (“I may not yet have the full explanation”) alongside relational and practical reliability (“I will take your symptoms seriously, explain what we do and do not know, agree adjustments that make today's encounter workable and safe, and ensure appropriate follow-up”). This is precisely where uncertainty tolerance becomes a professionalism milestone—demonstrated through respect, transparency, and dependable care—rather than a personal trait (5).

## From professional identity to implementation: education and system levers

For curriculum leads and educators, these competencies must be teachable and assessable, and they must be supported by system mechanisms that make reasonable adjustments reliable at the point of care. This should be operationalised as an education and professionalism intervention across undergraduate, postgraduate, and revalidation standards, using exercises explicitly designed to surface latent needs—for example, structured diagnostic and management pauses, brief equity reflections, and simulated consultations in which the “right” outcome is safe access and workable care under uncertainty rather than rapid diagnostic closure.

At system level, this requires agreed adjustments to be recorded once and implemented consistently across services, through mechanisms such as the Accessible Information Standard (AIS) and the Reasonable Adjustment Flag (RAF), with clear ownership, audit, and feedback loops when recorded adjustments are not delivered (9–12). Services should then reliably deliver what has been recorded: quiet rooms where feasible, alternative routes of access, flexible timetabling, predictable sequencing, and pacing-aware appointments, with processes that are responsive to variable needs without penalizing patients—for example, by avoiding rigid “Did Not Attend” penalties where sensory overload or access barriers have been recorded (9–12, 15).

To make the system honest, we must also measure what matters. Count how often patients with non-visible disabilities have active adjustment plans in operation (not merely recorded) and track safety- and outcome-related experience of care indicators, including whether patients report being asked about adjustments and whether documented adjustments were adhered to (18). Include non-visible disability in health inequalities dashboards so these groups are no longer overlooked (13). Digital prompts within electronic records may in future support implementation by reminding clinicians to ask about sensory or pacing requirements, but such tools should support—not replace—patient disclosure, clinical judgement, and reliable delivery of agreed adjustments.

Taken together, these are levers for professional identity, patient safety, and a practical agenda for curriculum development and evaluation. If effective care is defined only as rapid diagnostic closure, non-visible disability will remain systematically disadvantaged. If effective care is defined as safe access, credible listening, transparent communication of uncertainty, and reliable adjustments under uncertainty, medicine becomes both more equitable and more clinically accurate. The hidden curriculum can be revised by making uncertainty teachable, by rewarding pacing and co-construction in assessment, by embedding adjustments into routine systems so patients are not repeatedly forced to “prove” what cannot be seen, and by evaluating whether these educational and system changes translate into safer and more equitable care. Although the examples used in this paper are drawn mainly from the UK, the educational and clinical lessons are relevant across settings in which medicine privileges what is visible, measurable, and easily coded.
